# Geographical Distribution of E-cadherin Germline Mutations in the Context of Diffuse Gastric Cancer: A Systematic Review

**DOI:** 10.3390/cancers13061269

**Published:** 2021-03-12

**Authors:** Giovanni Corso, Federica Corso, Federica Bellerba, Patrícia Carneiro, Susana Seixas, Antonio Cioffi, Carlo La Vecchia, Francesca Magnoni, Bernardo Bonanni, Paolo Veronesi, Sara Gandini, Joana Figueiredo

**Affiliations:** 1 Division of Breast Surgery, European Institute of Oncology, Istituto di Ricovero e Cura a Carattere Scientifico (IRCCS), 20141 Milan, Italy; francesca.magnoni@ieo.it (F.M.); paolo.veronesi@ieo.it (P.V.); 2 Department of Oncology and Hemato-Oncology, University of Milan, 20122 Milan, Italy; 3 Department of Experimental Oncology, European Institute of Oncology, Istituto di Ricovero e Cura a Carattere Scientifico (IRCCS), 20141 Milan, Italy; federica.corso@ieo.it (F.C.); federica.bellerba@ieo.it (F.B.); sara.gandini@ieo.it (S.G.); 4 i3S-Instituto de Investigação e Inovação em Saúde, University of Porto, 4200-135 Porto, Portugal; pcarneiro@ipatimup.pt (P.C.); sseixas@ipatimup.pt (S.S.); jfigueiredo@ipatimup.pt (J.F.); 5 Institute of Molecular Pathology and Immunology of the University of Porto (IPATIMUP), 4200-135 Porto, Portugal; 6 Division of Urology, European Institute of Oncology, Istituto di Ricovero e Cura a Carattere Scientifico (IRCCS), 20141 Milan, Italy; antonio.cioffi@ieo.it; 7 Department of Clinical Sciences and Community Health, University of Milan, 20133 Milan, Italy; carlo.lavecchia@unimi.it; 8 Division of Cancer Prevention and Genetics, European Institute of Oncology, Istituto di Ricovero e Cura a Carattere Scientifico (IRCCS), 20141 Milan, Italy; bernardo.bonanni@ieo.it

**Keywords:** gastric cancer, E-cadherin, *CDH1* mutation, cancer geographical distribution

## Abstract

**Simple Summary:**

E-cadherin (*CDH1* gene) germline mutations are associated with the development of the autosomal cancer syndrome known as hereditary diffuse gastric cancer. About 30% of families fulfilling the clinical criteria established by the International Gastric Cancer Linkage Consortium have constitutional alterations of the *CDH1* gene. Different patterns of *CDH1* germline mutations have described as truncating, deletion, insertion, splice site, non sense, silence, and at last, missense alterations. The frequency of the different E-cadherin germline mutations in countries with different incidence rates for gastric carcinoma has reported extremely variable. In this study we aimed to assess the worldwide frequency of *CDH1* germline mutations in gastric cancers coming from different geographical areas, using a systematic approach.

**Abstract:**

Hereditary diffuse gastric cancer (HDGC) is a complex and multifactorial inherited cancer predisposition syndrome caused by *CDH1* germline mutations. Nevertheless, current *CDH1* genetic screening recommendations disregard an unbalanced worldwide distribution of *CDH1* variants, impacting testing efficacy and patient management. In this systematic review, we collected and analyzed all studies describing *CDH1* variants in gastric cancer patients originating from both high- and low-prevalence countries. Selected studies were categorized as family study, series study, and unknown study, according to the implementation of HDGC clinical criteria for genetic testing. Our results indicate that *CDH1* mutations are more frequently identified in gastric cancer low-incidence countries, and in the family study group that encompasses cases fulfilling criteria. Considering the type of *CDH1* alterations, we verified that the relative frequency of mutation types varies within study groups and geographical areas. In the series study, the missense variant frequency is higher in high-incidence areas of gastric cancer, when compared with non-missense mutations. However, application of variant scoring for putative relevance led to a strong reduction of *CDH1* variants conferring increased risk of gastric cancer. Herein, we demonstrate that criteria for *CDH1* genetic screening are critical for identification of individuals carrying mutations with clinical significance. Further, we propose that future guidelines for testing should consider GC incidence across geographical regions for improved surveillance programs and early diagnosis of disease.

## 1. Introduction

In the first half of the past century, GC was the most common cause of cancer-related deaths worldwide [1]. Although steadily declining, in 2017 GC remained the third cause of cancer-related deaths, after lung and colorectum, with almost over 850,000 deaths globally [2]. It was also the third cause of years of life lost (YLL), after lung and liver cancer [3]. A total of 1.22 million incident cases were estimated, with the highest incidence reported in Asia Pacific and East Asia, of which almost 50% were in China [3]. In the European Union, about 100,000 GC-related deaths were predicted for 2020 [4].

Almost two thirds of GC cases occur in developing countries with 42% in China alone [5]. In fact, the geographical distribution of GC is widely heterogeneous, with high-risk areas including East Asia (China, Japan and Korea), Eastern Europe, and parts of Central and South America [1,6,7,8,9]. Incidence rates are lower (<10 per 100,000 in men) in Southern Asia, North and East Africa, North America, Australia, and New Zealand [6]. Eastern Europe is the highest European risk area for GC with an incidence of 70,000 per year (Belarus area) [10]. Portugal and Italy also represent relevant European areas for stomach cancer prevalence, with incidence reports around 41,100 and 33,400 per year, respectively [11].

Widespread decline in GC incidence and mortality have been mainly associated to those of the corpus and pylori and have coincided with implementation of *Helicobacter pylori* (Hp) eradication programs, along with socio-economic improvements and advances in diet and food preservation. In contrast, cancer of the gastric cardia has increased in several high-income countries due to the increase in overweight and obesity, known etiological factors of gastro-esophageal reflux [12].

Although environmental risk factors account for variations in incidence and mortality rates worldwide, family history is a major risk factor for gastric cancer [13]. A number of genetic loci have been associated with GC risk, which may directly impact disease progression or interact with environmental factors in the causal pathway [1,14].

GC presents familial aggregation in about 10%, and only 3% shows a clear inherited cancer predisposition, so-called “hereditary”, associated with a documented germline mutation. Among these, germline defects in *CDH1*, encoding the epithelial cadherin, have been particularly explored in the context of both familial and sporadic gastric cancer development [15].

The first description of *CDH1* germline mutations was reported in Maori kindred and families with diffuse gastric cancer (DGC) and lobular breast cancer (LBC) aggregation [16]. In 1999, the International Gastric Cancer Linkage Consortium (IGCLC) defined the hereditary diffuse gastric cancer (HDGC) syndrome and established clinical criteria for *CDH1* genetic screening of individuals and families at risk [17]. Using those first guidelines, the detection rate of *CDH1* mutations was approximately 40% in individuals fulfilling the clinical criteria [18]. However, the guidelines were subsequently revised given that *CDH1* germline mutations were also identified in individuals who did not meet testing criteria [19,20,21]. Hansford and colleagues reported that in individuals meeting the IGCLC 2010 criteria and with *CDH1* germline mutation [20], the cumulative lifetime GC risk at 80 years of age was 70% (95% CI, 59–80%) for males and 56% (95% CI, 44–69%) for females, whereas breast cancer lifetime risk for females was 42% (95% CI, 23–68%) [22].

To date, more than 155 *CDH1* mutations affecting the entire coding sequence and functional domains of E-cadherin have been identified in the context of HDGC [20,22]. Whereas the majority of HDGC patients display *CDH1* truncating mutations that induce a deleterious effect and are thus a bona fide DGC cause, around 20% harbor mutations of the missense type, which represent a major clinical challenge [20] Indeed, missense variants are difficult to assess phenotypically, thus leading to critical issues concerning genetic counseling and clinical management. Further, their incomplete penetrance masks their identification and classification, contributing to variant dissemination among populations [23]. Importantly, failure to incorporate information and ascertain pathogenicity of missense variants perpetuates misestimating of *CDH1* penetrance and the diagnostic dilemma surrounding affected families. In an era of high-throughput genome sequencing and multiplex gene panel testing, this problem is becoming unwieldy with the identification of an increasing number of variants of unknown significance (VUS), not only in disease but also in individuals without family history of gastric cancer [24,25] This, along with the fact that the majority of cancer screening programs do not recommend *CDH1* testing in the absence of specific clinical criteria, urges the need to streamline *CDH1* screening. More so, there is a lack of systematic results regarding *CDH1* genetic screening across countries, creating a void in terms of mutation geographic distribution [26]. This has prompted us to perform a comprehensive evaluation of germline mutations associated to HDGC, which may explain the large variability in GC epidemiology and provide insights to define priorities for effective screening and improved management.

## 2. Methods

### 2.1. Study Accuracy and Selection

**Figure 1:**
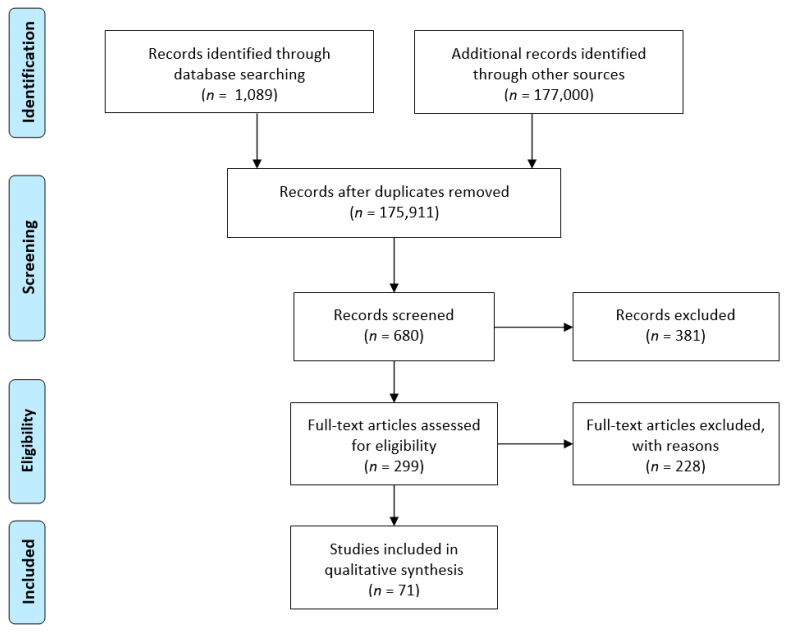
Scheme illustrating the study design. Articles nr. 228 were excluded if not original (reviews), were reports of somatic studies (not germline), or not associated with gastric cancer (i.e., lobular breast, ovarian, colon, cancers).

Accuracy of this systematic review was assessed using the checklist of items in accordance with the Preferred Reporting Items for Systematic reviews and Meta-Analyses (PRISMA) statement. In particular, we organized this review as following: (a) Identification: to assess the *CDH1* germline mutation frequency in the world; (b) Screening: to search all identified *CDH1* germline mutations in DGC patients and in asymptomatic carriers; (c) Eligibility: to include all data reported in an accessible source bank (the National Library of Medicine’s MEDLINE database); (d) Inclusion: to analyze the obtained results with a study of frequencies (Figure 1).

We revised all *CDH1* germline mutations reported in MEDLINE from 1998 to November 2019, including original reports and literature reviews edited in English language. For search of available literature, the following terms were used: E-cadherin; *CDH1* gene; germline mutation; genetic screening; HDGC; IGCLC; familial GC; diffuse histotype; Maori kindred; and prophylactic gastrectomy.

The analysis was limited to studies involving subjects affected by HDGC, early-onset gastric cancer (EOGC), and unselected GC patients screened for *CDH1* germline mutations, in which at least one likely pathogenic, VUS or pathogenic *CDH1* variant was identified. These studies were subsequently categorized into three categories: series study, family study and unknown study, depending on whether or not they fulfilled clinical criteria for genetic testing.

### 2.2. Group Description

The series study group included 31 studies from populations screened for *CDH1* germline mutations, comprising only individuals affected by GC. The authors analysed a consecutive series of GC population, but members of these individuals were not screened. Patients were grouped according to the inclusion of clinical criteria established by the IGCLC [27]: no criteria GC patients (11 studies); patients fulfilling HDGC criteria (15 studies); and early onset GC patients (five studies). The early onset GC subset encompassed cases of sporadic GC with age at diagnosis of less than 45 years old. In the series study only individuals affected by GC were analysed for *CDH1* germline mutations.

The family study group comprised 36 studies of families strictly fulfilling the IGCLC criteria. In those studies, genetic screening was performed both in GC patients and asymptomatic individuals.

Finally, the unknown study group (five studies) included individuals for whom familial information regarding cancer phenotypes is lacking.

### 2.3. Data Extraction, Statistical Methods and Quality Assessment of Studies

For the series study group, information on country of origin (or ethnicity, when origin was not available), type of mutation, ClinVar/LOVD classification, total number of subjects screened for *CDH1* mutations and total number of *CDH1* mutation carriers was retrieved (Table S1) [19,22,28,29,30,31,32,33,34,35,36,37,38,39,40,41,42,43,44,45,46,47,48,49,50,51,52,53,54,55,56]. When available, mean age of all subjects screened for *CDH1* mutations and mean age of *CDH1* mutation carriers was calculated.

For the family study articles, we collected data on country (or ethnicity), mutation type and classification, age of proband, total number of screened subjects harbouring *CDH1* mutations, number of carriers affected by gastric cancer, and number of carriers affected by other cancer types (Table S2) [18,37,57,58,59,60,61,62,63,64,65,66,67,68,69,70,71,72,73,74,75,76,77,78,79,80,81,82,83,84,85,86,87,88,89]. Mean age of *CDH1* mutation carriers affected by gastric cancer and mean age of carriers affected by other cancer types was also determined.

In the unknown study, we have explored data on country of origin (or ethnicity), as well as mutation type and classification (Table S3) [17,20,22,78,90,91].

Descriptive statistics (absolute and relative frequencies) are presented to describe frequencies of *CDH1* mutations and total number of *CDH1* mutation carriers by type of study design and countries and differences in frequencies are compared with Chi-square tests. All tests were two-sided and statistical significance was set at *p*-value < 0.05.

### 2.4. Missense Variant Scoring

The potential effect of *CDH1* missense variants was scored considering ClinVar variant interpretation (available at https://www.ncbi.nlm.nih.gov/clinvar/ accessed on 6 November 2020), in silico predictions and functional in vitro assays. Variants that were not submitted to ClinVar were investigated at LOVD database (https://www.lovd.nl/ accessed on 6 November 2020). For in silico prediction of variant pathogenicity, scores from 20 algorithms were retrieved from Ensembl Variant Effect Predictor (VEP) web interface (https://www.ensembl.org/Tools/VEP accessed on 6 November 2020), using GRCh37 assembly and *CDH1* gene (localization GRCh37:CM000678.1). Scores evaluated include CADD, DANN, Eigen-PC, FATHMM, GERP++, LRT, M-CAP, MetaLR, MetaSVM, MutPred, MutationAssessor, MutationTaster, PROVEAN, Polyphen2_HDIV, Polyphen2_HVAR, REVEL, SIFT, SiPhy_29way_logOdds, fathmm-MKL and phyloP100way_vertebrate. Ranked scores (varying from 0 to 1) were selected to allow a normalization of scales across predictive tools. Subsequently, ranked scores were averaged to provide a single and unbiased value combining the distinct methodological approaches of each in silico tool. Published data from in vitro functional tests, including aggregation and matrigel invasion assays, were collected. Variant score was attributed as following: Benign/Retains function/Tolerated is represented as −−−; Pathogenic/Loss of function/Damaging as +++; Likely pathogenic/Possibly damaging as ++/−; Likely benign/Possibly tolerated as +/−−; VUS/Inconclusive as +/−; and Not submitted/Not studied as 0. The percentage of + frequency was calculated and variants with values > 50% were considered relevant, whereas variants below 50% were considered not relevant. Variants with + frequency of 50% were categorized as undetermined.

## 3. Results

### 3.1. Study Features

To perform a comprehensive analysis of *CDH1* alterations and their correlation with GC incidence, we have first collected and assembled reports into series study, family study and unknown study groups, according to the compliance or not of HDGC criteria for *CDH1* genetic screening. Among the series study group, a total of 2.547 primary GCs were screened for *CDH1* germline mutations, identifying 187 germline alterations (7.2%). Specifically, 52 (4.2%) *CDH1* mutations were identified in 1.224 GC patients from no criteria studies, 113 in 653 (17.3%) GCs from HDGC criteria studies, and 22 in 670 (3.2%) early onset GCs. Regarding the mutation type, we have identified nine deletions, one insertion, 29 missense, three non-sense and 10 splice site mutations in no criteria studies. In HDGC criteria studies, we have detected 36 deletions, nine insertions, 19 missense, 31 non-sense and 18 splice site alterations. Finally, in early onset GCs, the distribution was as following: one deletion, zero insertions, six missense (27.3%), two non-sense and 13 splice sites (Table S1).

In the family study group, 356 *CDH1* germline mutations were detected, including 225 in patients affected by GC (63.2%) and 131 in asymptomatic carriers (36.8%). The mean age of proband affected by primary GC (*CDH1* mutation positive) was 38.4 years old, and 40.1 in the remaining GC patients identified as carriers of *CDH1* mutations. Additionally, 20 *CDH1* alterations were identified in patients diagnosed with other primary cancers, namely 10 mutations in breast (mean age at diagnosis 49.6 years old), six in colon (mean age at diagnosis 51.1 years old), two in abdominal carcinomatosis, one in tongue, and one in prostate cancer.

Interestingly, regarding missense mutations, the GC probands displayed a mean age of 36.9 years old, and within a total of 71 alterations identified, 30 (42.3%) were found in asymptomatic carriers. In the non-missense mutation group, the mean age of the GC proband was 38.8 years old, and 101 (101/285, 35.4%) were GC-negative (Table S2).

In the unknown study group we identified a total of 20 *CDH1* germline mutations (Table S3).

### 3.2. Mutation Type Frequencies

**Figure 2:**
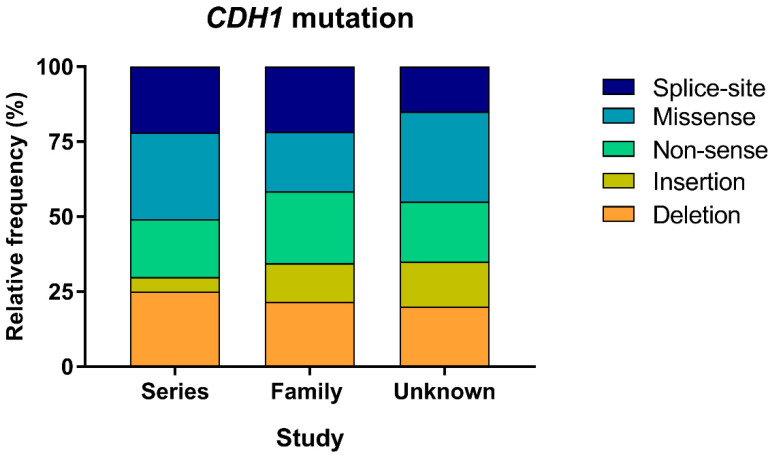
Relative frequency of *CDH1* mutation types. For each study group, the relative frequency of deletions, insertions, as well as non-sense, missense and splice-site alterations is represented in the graph. Percentages were calculated based on the total number of mutations found in each study group. The series study group includes 187 mutations, the family study one 356, and the unknown study group encompasses 20 mutations.

A total of 563 *CDH1* germline mutations were identified, with the vast majority detected in the family study group (356/563, 63%). Type of mutation is significantly associated with study groups (*p* = 0.05). In the series study group 187 (33.2%) mutations were registered, whereas only 20 (3.6%) mutations were reported in the unknown study group (Table 1). Overall, missense variants represent 23.3% of the cases, followed by deletions (22.6%), non-sense (22.2%), and splice-site alterations (21.5%). Insertions are less frequently described, constituting 10.3% of all *CDH1* mutations. However, the relative frequency of mutation types varies across the different study groups (Figure 2). In particular, in the family study group, non-sense mutations represent the most frequently identified alterations (23.9%), with deletions and splice-site alterations accounting for 21.6% each. In the series study and unknown study groups, missense variants comprise the largest subsetting, reaching respectively 28.9% and 30%, in contrast to the 19.9% observed in the family study group. Deletions are the second most frequent mutation type detected in both the series and unknown studies groups (24.6% and 20.0%, respectively). Non-sense variants are found in 19.3% of carriers included in the series study group and in 20% of cases from the unknown group.

These results suggest that different mutation types may reflect distinct GC management and surveillance programs. More so, it is not surprising that truncating mutations (non-sense, deletions and splice-site) are promptly diagnosed given their severe and possibly more penetrant effect. In contrast, missense variants may retain residual E-cadherin activity, producing less striking familiar cancer aggregation and, consequently, impairing their predictive value as drivers of HDGC.

### 3.3. Geographical Distribution of CDH1 Mutations

**Figure 3:**
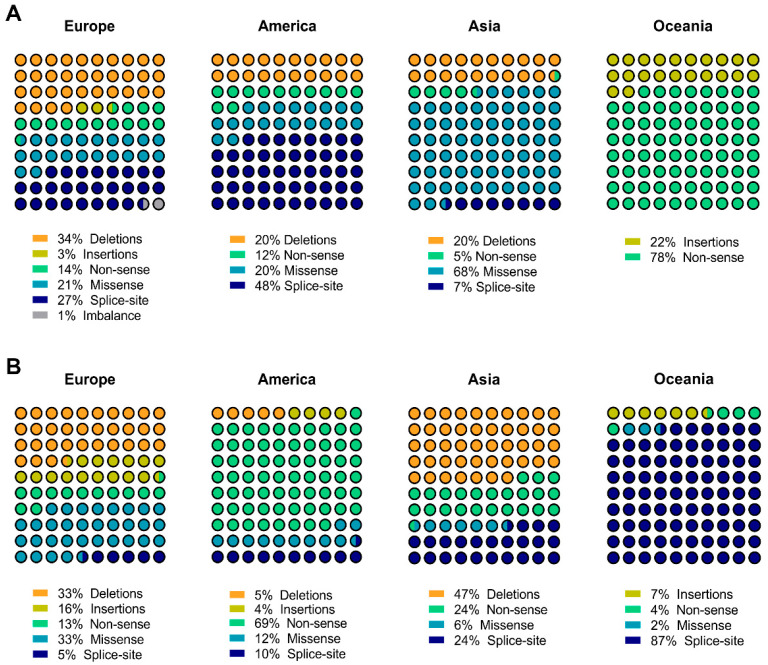
*CDH1* mutation worldwide distribution. Representation of relative frequencies of the different *CDH1* mutation types within the European, American, Asian and Oceania continents, in the settings of either the series study (**A**) or the family study (**B**) groups.

Given that prevalence of gastric cancer is not homogenous worldwide, we next evaluated the geographical distribution of mutations collected in the series and family studies groups. For this purpose, we grouped mutations with known country of origin by continent. The unknown study group was not included in this analysis since we lacked information on country of origin in 55% of alterations, and the remaining mutations were collected from only three reports, which could potentially result in a bias. In the series study group, we verified that 45.5% (71/156) of alterations were detected in individuals from European origin, with lower percentages identified in Asian and American individuals (26.3 and 16.0%, respectively), as well as in Oceania (11.5%). Remarkably, we verified that the predominant mutation type varies across geographical regions. As depicted in Figure 3A, deletions are more frequent in Europe (34%), splice-site in America (48%), missense in Asia (68%), and non-sense in Oceania (78%). The high prevalence of missense mutations in Asia is mainly attributed to Korean and Japanese populations.

Despite obvious differences in the number of mutations reported in each setting (series or family study), we demonstrate that, in the family study context, 51.9% (150/289) of mutations are of European origin, 26.6% (77/289) of American, 15.6% (45/289) of Oceania and 5.9% (17/289) of Asian origins. In this group, a distinct distribution pattern of mutation classes was also observed worldwide (Figure 3B). Deletions (33%) and missense (33%) alterations are the most common alteration type in Europe, non-sense in America (69%), deletions in Asia (47%), and splice-site in Oceania (87%). A striking difference was found in Europe, where the relative frequency of splice-site alterations decreases from series to family studies, while the missense category increases. Likewise, in the American continent, splice-site alterations decrease from 48% in the series study group to 10% in the family group, which in turn depicts a substantial increase of non-sense mutations. The opposite effect is verified in Oceania: splice-site alterations, that were not identified in the series study, appear in 87% of the family study subjects; whereas the non-sense relative frequency decreases from 78% to 4%. Another difference is detected at the Asian region and involves missense variants, where a very high frequency of these variants (78%) occurs in the series study but not in the family study group. This could be a result of low penetrance of missense variants, in turn leading to their underestimation and, consequently, segregation within populations. We observe that, along with other host genetic and environmental factors, *CDH1* missense variants are associated to the high incidence of gastric cancer in Asian populations.

### 3.4. Missense Variant Relevance across Geographical Regions and Study Contexts

To further evaluate the impact of *CDH1* missense variants and establish potential associations with population distribution, we have collected data from ClinVar classification, as well as from in vitro experimental evidence and in silico predictions, which are currently not considered by the ACMG/AMP variant curation guidelines.

From the 42 missense variants gathered, 14 (33%) were classified by ClinVar as VUS. However, upon application of a combined score system, we unveiled useful information for a possible categorization in 13 out of the 14 variants (Table 2). Overall, we have categorized 41 (out of 42) missense variants: 15 as not relevant and 26 as relevant variants. With this approach, we were able to provide insights on the relevance of variants that would remain unclassified or are classified disregarding in vitro and in silico data, according to just ClinVar criteria. Unfortunately, no in vitro studies were published concerning the effect of 14 missense variants, impairing a more accurate grading.

**Figure 4:**
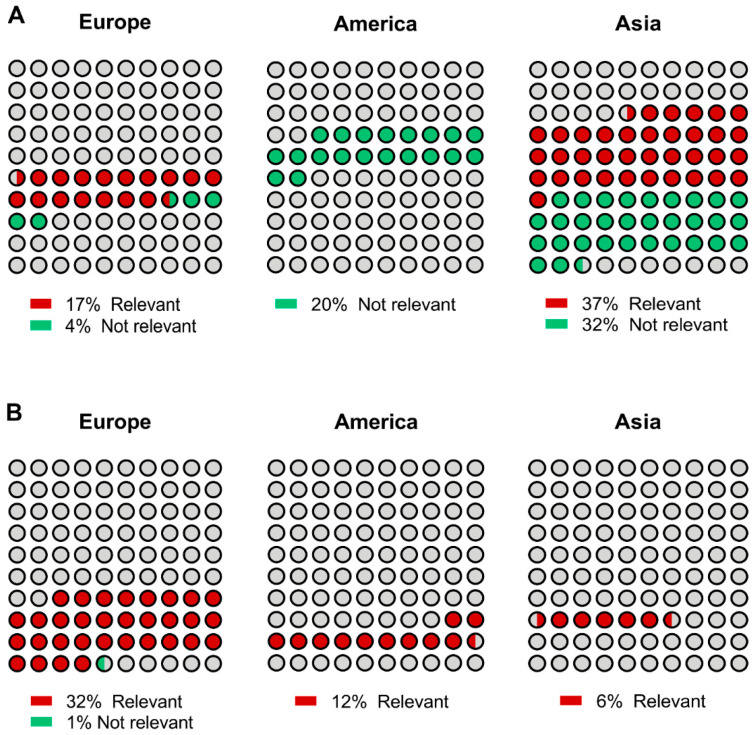
*CDH1* missense mutation distribution based upon variant scoring. The relative frequency of the different missense variant categories was evaluated in continents displaying higher missense variant frequencies. (**A**) Series study, (**B**) Family study.

Having established a comprehensive score for missense variant categorization, we next investigated its significance on the different geographical areas and study groups. As observed in Figure 4, with the exception of 1 variant, missense alterations reported in the family study group (59/60) were considered relevant.

A very distinct situation was observed in the series study context, particularly, in those variants identified in American and Asian individuals. In fact, all variants with American origin were scored as not relevant, and from those reported in Asia (*n* = 28), 15 were relevant and 13 were categorized as non-relevant.

Within the series study group, missense mutations occur as sparse events in countries with low-incidence for GC, such as USA, New Zealand, France, Canada, and UK, but are frequent in Korea, Japan, China and Italy, which are high-incidence countries (9% vs. 51% respectively, *p*-value < 0.001 Chi-square test)

Overall, it is clear that selection based upon family criteria identifies individuals carrying mutations with clinical relevance. On the other hand, unbiased screening may lead to an over selection of individuals harboring *CDH1* variants that, certainly, do not award increased risk of developing gastric cancer.

## 4. Discussion

GC remains one of the most common malignancies worldwide and the fifth leading cause of cancer-related mortality, despite reported significant declines over the past 50 years. Not surprisingly, decreased GC mortality has been largely associated to a trending decrease in incidence. Progress in survival rates over the past few decades has been limited to western countries, with overall five-year survival around or below 30%. Strikingly, in Japan and Korea, historically high incidence countries, five-year survival is now over 60%, which has been attributed to their extensive screening and early diagnosis programs [92].

Family history also accounts for GC incidence variations worldwide, with rates ranging from 2.8% in Sweden [93] to 36.6% in Japan [94]. Identification of germline inactivating mutations of E-cadherin were the first evidence of a clear molecular basis for familial GC susceptibility [16]. The association of germline *CDH1* mutations and HDGC predisposition was a breakthrough that sparked the establishment of specific guidelines for genetic screening and surveillance of patients who are at high risk of developing GC [27] However, current *CDH1* genetic testing guidelines neglect worldwide distribution of variants, in part because such information has not been carefully curated. Furthermore, identification of unexpected *CDH1* due to the increasing use of multigene panels poses a challenge in GC management. As such, we found it crucial to compile a systematic review encompassing all reports of *CDH1* variants described in GC patients and analyze mutation profiles and worldwide incidence with respect to implementation of HDGC clinical criteria for genetic testing. Information about family history for GC remains fundamental to plan a genetic test and to adopt strategic intervention aiming to reduce the GC risk in asymptomatic individuals.

In accord with some recent literature data, we observed that the cumulative incidence of GC within *CDH1* germline mutations is decreasing. Xicola et al. reported that the overall cumulative risk of GC by age 80 in families with at least one GC was about 37% among men and 24% among women [95]. Also, Roberts described that the cumulative incidence of GC at age 80 years was 42% (95% CI, 30–56%) for men and 33% (95% CI, 21–43%) for women with pathogenic variants in *CDH1* [96]. Both studies were a review of already published families, and not original analysis, as reported in Hansford et al. [22].

A key finding of this review is that, overall, screened GC patients depicted a low frequency of *CDH1* germline mutations (about 7% in 2587 GCs). Importantly, the probability to identify a *CDH1* mutation with clinical relevance increased when individuals were selected based upon clinical criteria for HDGC screening (17.3% in cases fulfilling HDGC criteria vs. 4% in unselected cases).

According to our analysis, overall GC frequency with familial clustering was 23.9% vs. 11.9 in high- and low-risk areas, respectively. Interestingly, we could determine that the frequency of *CDH1* germline mutations is not directly related with the incidence of familial gastric carcinoma. In fact, in the family study group, we could observe a high number of mutations detected in North America and a lower number in Asia. In support of these results, other studies have proposed that in high-risk areas, aggregation of GC cases is probably associated with environmental and cultural factors, including dietary habits [97,98]. Further, other reports describe that genetic factors, such as *CDH1* germline alterations, are rarely identified in high-risk areas [56,99]. Nonetheless, it should not be disregarded that these outcomes may reflect distinct resources and screening programs across countries. Geographical variability represents a risk factor for gastric carcinoma. We verified in this study that *CDH1* germline mutations in GC middle/high-risk area are rather rarely identified respecting to low-risk area; and in middle/high-risk area the probability to perform a *CDH1* genetic screening with negative result is high enough, in which maybe operates environmental factors. Diet and high consumption of specific foods are associated positively with GC risk, in particular with the intestinal sub-type.

Regarding mutation type, we determined that missense, deletions, non-sense and splice-site alterations present similar overall frequencies (23.3–21.5%). More so, mean age at GC diagnosis was similar in carriers of both missense and truncating *CDH1* mutations. However, 42% of missense mutation carriers were asymptomatic, whereas a lower percentage of truncating mutation carriers (35%) was unaffected by GC. This can be partly due to the lower penetrance of missense mutations as a result of residual activity retained by these E-cadherin molecules encompassing an amino acid substitution [100,101]. The pleiotropic effect of *CDH1* mutations can also play a role in this context. Recent evidence has emerged demonstrating that *CDH1* mutations may result in lobular breast cancer and in several congenital abnormalities, without personal or family history of GC [102]. Unfortunately, incorporation of information regarding clinical history of missense mutation carriers remains scarce hampering ascertainment of missense mutation pathogenicity and perpetuating the diagnostic dilemma surrounding affected families.

Within the series study group, missense mutations occur as sparse events in countries with low-incidence for GC, such as USA, New Zealand, France, Canada, and UK, but are frequent in Korea, Japan and Italy, which are high-incidence countries. This suggests that missense mutations exert a less aggressive effect in family phenotypic genealogy, consequently leading to their concealment and segregation at very low frequencies in the general population. It is of note that evaluation of putative relevance of missense mutations revealed that only 53% of those reported in Asia are clinically different. In America, the impact of mutation categorization was even more striking with 100% of mutations scored as not relevant. In the family study population, we verified that the contribution of non-sense, deletions and splice-site alterations is indisputable, despite the huge difference in the type of mutations across continents. In this context, the frequency of missense mutations varies from 2% in Oceania to 33% in Europe. Noteworthy, in 98% of these cases, a relevant effect was estimated.

This study presents a limitation in the method that should be considered. The *CDH1* germline mutation frequency could be affected by research activity, as publication bias or access to medical care, considering that some *CDH1* mutations could be unpublished in MEDLINE. However, MEDLINE is the unique search engine in which we can access easily and freely and unfortunately, we do not have access in confidential as well in unpublished data. This bias should be very small, because we carefully collected all *CDH1* mutations recorded in MEDLINE, and all collected data were revised from four independent Authors.

## 5. Conclusions

In conclusion, our results demonstrate that *CDH1* mutations are more frequently identified in countries with low-incidence for GC, and that the application of criteria for genetic screening is critical for a higher detection rate, as well as for the identification of mutations with proven clinical relevance. This systematic analysis clearly corroborates that following guidelines for screening and surveillance of patients at high risk has the potential to diagnose and treat GC at an earlier stage, improving survival rates. In the absence of clinical criteria and of familial genotype-phenotype correlation, detection of a *CDH1* mutation imposes a clinical management issue given that the consensual risk-reducing recommendation regarding DGC is a radical and life-changing gastrectomy. It is our recommendation that those individuals should be closely monitored through an intense surveillance program, which should both contribute to early diagnosis and to enlighten disease etiology.

## Figures and Tables

**Table 1:** *CDH1* mutation types identified within study groups.

Mutation Type	Series Study	Family Study	Unknown Study	Total	*p*-Value
Deletion	46 (24.6%)	77 (21.6%)	4 (20.0%)	127 (22.6%)	0.05
Insertion	9 (4.8%)	46 (12.9%)	3 (15.0%)	58 (10.3%)	-
Non-sense	36 (19.3%)	85 (23.9%)	4 (20.0%)	125 (22.2%)	-
Missense	54 (28.9%)	71 (19.9%)	6 (30.0%)	131 (23.3%)	-
Splice-site	41 (21.9%)	77 (21.6)	3 (15.0%)	121 (21.5%)	-
Imbalance	1 (0.5%)	0 (0.0%)	0 (0.0%)	1 (0.2%)	-
Total	187 (33.2%)	356 (63.2%)	20 (3.6%)	563	-

*p*-value from Chi-square excluding Imbalance mutation type.

**Table 2:** Potential impact of missense variants.

HGVS	Protein Change	ClinVar Classification	In Vitro	In Silico	Total +	Total −	+ Frequency%	Variant Relevance
2T>C	M1T	+++	0	++/−	5	1	83	Yes
3G>C	M1I	+++ ^A^	0	++/−	5	1	83	Yes
3G>A	M1I	+++	0	++/−	5	1	83	Yes
48G>C	Q16H	0	+++	++/−	5	1	83	Yes
79C>T	P27S	+/−	0	+/−−	2	3	40	No
185G>T	G62V	+/−	+++	++/−	6	2	75	Yes
286A>G	I96V	+/−−	0	+/−−	2	4	33	No
313T>A	S105T	+/−	0	+/−−	2	3	40	No
353C>G	T118R	+/−	+++	−−−	4	4	50	Unknown
387G>T	Q129H	+/−−	0	−−−	1	5	17	No
554A>T	E185V	+/−	---	++/−	3	5	38	No
604G>A	V202I	−−−	0	+/−−	1	5	17	No
641T>C	L214P	+++ ^A^	+++	+++	9	0	100	Yes
695C>G	S232C	+/−	---	++/−	3	5	38	No
715G>A	G239R	++/−	+++	++/−	7	2	78	Yes
731A>G	D244G	+/−	0	++/−	3	2	60	Yes
820G>A	G274S	+/−−	---	+/−−	2	7	22	No
892G>A	A298T	−−−	+++	++/−	5	4	56	Yes
977T>A	I326N	0	+++	+++	6	0	100	Yes
1018A>G	T340A	−−−	+++	−−−	3	6	33	No
1118C>T	P373L	+/−−	+++	+++	7	2	78	Yes
1225T>C	W409R	+/−−	+++	+++	7	2	78	Yes
1243A>C	I415L	+/−	+/-	+/−−	3	4	43	No
1285C>T	P429S	+/−	+++	++/−	6	2	75	Yes
1409C>T	T470I	−−−	+++	+++	6	3	67	Yes
1460T>C	V487G	+++ ^A^	0	+/−−	4	2	67	Yes
1676G>A	S559N	+/−	0	+/−−	2	3	40	No
1679C>G	T560R	+++	+++	++/−	8	1	89	Yes
1748T>G	L583R	+++ ^A^	+++	++/−	8	1	89	Yes
1774G>A	A592T	−−−	−−−	++/−	2	7	22	No
1806C>A	F602L	0	0	+/−−	1	2	33	No
1849G>A	A617T	−−−	−−−	+/−−	1	8	11	No
1888C>G	L630V	−−−	0	++/−	2	4	33	No
1901C>T	A634V	++/−	+++	+/−−	6	3	67	Yes
2195G>A	R732Q	++/−	+++	++/−	7	2	78	Yes
2245C>T	R749W	+/−	+++	+++	7	1	88	Yes
2248G>A	D750N	+/−	+/−	+++	5	2	71	Yes
2315T>A	L772Q	0	0	+++	3	0	100	Yes
2343A>T	E781D	+/−	+++	+/−−	5	3	63	Yes
2396C>G	P799R	+/−	+++	+++	7	1	88	Yes
2413G>A	D805N	−−−	+++	+++	6	3	67	Yes
2494G>A	V832M	−−−	+++	+++	6	3	67	Yes

To determine the relevance of missense variants described in this study, a classification score was implemented according to several parameters, including ClinVar interpretation, available in vitro experimental evidence and in silico predictions. In vitro experiments are based upon cell-cell aggregation and invasion assays, as well as E-cadherin expression and localization studies in cell lines transfected with the different variants. An in silico score was established by combining a set of 20 predictive algorithms. Score was attributed as following: Benign/Retains function/Tolerated is represented as −−−; Pathogenic/Loss of function/Damaging as +++; Likely pathogenic/Possibly damaging as ++/−; Likely benign/Possibly tolerated as +/−−; VUS/Inconclusive as +/−; and Not submitted/Not studied as 0. ^A^ indicates variants not submitted to ClinVar and whose classification is based upon LOVD database.

## Data Availability

Data is contained within the article or Supplementary Materials.

## Supplementary Materials

The following are available online at https://www.mdpi.com/2072-6694/13/6/1269/s1, Table S1. *CDH1* germline mutations identified in Series Study group, Table S2. *CDH1* germline mutations identified in Family Study group), Table S3. *CDH1* germline mutations identified in Unknown Study group (* Unpublished data).
