# Supplementary material for: Geographical Distribution of E-cadherin Germline Mutations in the Context of Diffuse Gastric Cancer: A Systematic Review
Source: Cancers (Basel). 2021 Mar 12;13(6):1269. doi: 10.3390/cancers13061269 (PMC8001745; doi:10.3390/cancers13061269)
Supplement: Supplementary file 1 [file cancers-13-01269-s001.pdf]

Supplementary Materials: Geographical Distribution of E-cadherin Germline Mutations in the Context of Diffuse Gastric Cancer: A Systematic Review

Giovanni Corso, Federica Corso, Federica Bellerba, Patrícia Carneiro, Susana Seixas, Antonio Cioffi, Carlo La Vecchia, Francesca Magnoni, Bernardo Bonanni, Paolo Veronesi, Sara Gandini and Joana Figueiredo

Table S1. *CDH1* germline mutations identified in Series Study group.

First Author	Country	Type	HGVS	Protein Change	ClinVar/LOVD Classification	In Vitro Data	SIFT	PolyPhen-2	Mean Age Carriers	Carriers	Mean Age Screened	Screened	Criteria
Shin [29]	Korea	Missense	2494G>A	V832M	Benign	Function loss	Damaging	Probably damaging	60	7	64	305	Unselected
Guindalini [30]	Brasil	Missense	313T>A	S105T	VUS	Not studied	Tolerated	Benign	41	1	39	88	EOGG
	Brasil	Missense	387G>T	Q129H	Not submitted	Not studied	Tolerated	Benign	-	1	-	-	-
	Brasil	Missense	1676G>A	S559N	Not submitted	Not studied	Tolerated	Benign	-	1	-	-	-
	Brasil	Missense	1806C>A	F602L	Not submitted	Not studied	Tolerated	Benign	-	1	-	-	-
Kim [31]	Korea	Missense	2494G>A	V832M	Benign	Function loss	Damaging	Probably damaging	37	2	70	107	Unselected
Hakkaart [32]	New Zealand	Non sense	190C>T	-	Pathogenic	-	-	-	33	4	56	94	HDGC
	New Zealand	Non sense	1792C>T	-	Pathogenic	-	-	-	-	4	-	-	-
	New Zealand	Non sense	2195G>A	-	Likely pathogenic	-	-	-	-	1	-	-	-
	New Zealand	Non sense	2287G>T	-	Pathogenic/Likely pathogenic	-	-	-	-	4	-	-	-
	New Zealand	Insertion	2382insC	-	Pathogenic	-	-	-	-	4	-	-	-
Tedaldi [33]	Italy	Deletion	1-?_163+?del	-	Not submitted	-	-	-	41	1	?	79	HDGC
	Italy	Non sense	308G>A	-	Pathogenic	-	-	-	-	1	-	-	-
	Italy	Deletion	360delG	-	Pathogenic	-	-	-	-	1	-	-	-
	Italy	Non sense	781G>T	-	Likely pathogenic	-	-	-	-	2	-	-	-
	Italy	Non sense	1003C>T	-	Pathogenic	-	-	-	-	1	-	-	-
	Italy	Non sense	1137G>A	-	Pathogenic	-	-	-	-	1	-	-	-
	Italy	Deletion	1965delG	-	Not submitted	-	-	-	-	1	-	-	-
	Italy	Deletion	2114delT	-	Not submitted	-	-	-	-	1	-	-	-
Molinaro [34]	Italy	Splice site	688-1G>C	-	Not submitted	-	-	-	39	1	42	30	HDGC
	Italy	Missense	2315T>A	L772Q	Not submitted	Not studied	Damaging	Probably damaging	-	1	-	-	-
	Italy	Deletion	833-476_1138-463del	-	Not submitted	-	-	-	-	1	-	-	-
	Italy	Imbalance	Allelic Imbalance	-	Not submitted	-	-	-	-	1	-	-	-
	Italy	Non sense	187C>T	-	Pathogenic	-	-	-	-	1	-	-	-
	Italy	Missense	1901C>T	A634V	Likely pathogenic	Function loss	Tolerated	Benign	-	1	-	-	-

Garziera [35]	Italy	Splice site	-71C>G	-	Benign/Likely benign	-	-	-	?	3	61	59	Unselected
	Italy	Missense	820G>A	G274S	Likely benign	Retains function	Damaging	Probably damaging	-	1	-	-	-
	Italy	Missense	892G>A	A298T	Benign	Function loss	Damaging	Probably damaging	-	1	-	-	-
	Italy	Missense	1409C>T	T470I	Benign	Function loss*	Damaging	Probably damaging	-	1	-	-	-
	Italy	Splice site	1937-13T>C	-	Benign	-	-	-	-	2	-	-	-
Garziera [35]	Italy	Missense	1774G>A	A592T	Benign	Retains function	Damaging	Possibly damaging	-	1	-	-	-
Bacani [36]	Canada	Splice site	-117G>A	-	Not submitted	-	-	-	?	1	<51	58	EOGC
	Canada	Splice site	-71C>G	-	Not submitted	-	-	-	-	2	-	58	-
	Canada	Deletion	41delT	-	Pathogenic	-	-	-	-	1	-	59	-
	Canada	Splice site	48+5C>G	-	Not submitted	-	-	-	-	1	-	58	-
	Canada	Splice site	48+15C>G	-	Not submitted	-	-	-	-	2	-	58	-
	Canada	Splice site	387+26C>T	-	Not submitted	-	-	-	-	1	-	76	-
	Canada	Splice site	1937+13T>C	-	Benign	-	-	-	-	2	-	27	-
	Canada	Splice site	2295+53G>A	-	VUS	-	-	-	-	1	-	76	-
Corso [37]	Italy	Splice site	-63C>A	-	Not submitted	-	-	-	42	1	42	21	EOGC
Oliveira [38]	North Europa	Deletion	Del exon 1–2	-	Not submitted	-	-	-	36	1	?	93	HDGC
	Canada	Deletion	Del exon 1–2	-	Not submitted	-	-	-	-	1	-	-	-
	Eastern Europa	Deletion	Del exon 1–2	-	Not submitted	-	-	-	-	1	-	-	-
	Southern Europa	Deletion	Del 50-UTR-exon 1	-	Not submitted	-	-	-	-	1	-	-	-
	Central Europa	Deletion	Del exon 14–16	-	Not submitted	-	-	-	-	1	-	-	-
	Central Europa	Deletion	Del exon 16	-	Not submitted	-	-	-	-	1	-	-	-
Suriano [39]	Unknown	Missense	3G>C	M1I	Pathogenic	Not studied	Damaging	Benign	?	1	?	31	HDGC
	Unknown	Deletion	1063del	-	Pathogenic	-	-	-	-	1	-	-	-
	Unknown	Non sense	187C>T	-	Pathogenic	-	-	-	-	1	-	-	-
	Unknown	Non sense	1792C>T	-	Pathogenic	-	-	-	-	1	-	-	-
	Hispanic	Splice site	2161C>G	-	VUS	-	-	-	-	1	-	-	-
	Unknown	Non sense	1003C>T	-	VUS	-	-	-	-	2	-	-	-
	Unknown	Deletion	2276delG	-	Pathogenic	-	-	-	-	1	-	-	-
	Caucasian	Missense	1285C>T	P429S	Pathogenic	Function loss	Damaging	Probably damaging	-	1	-	-	-
Chen [40]	China	Deletion	44_46delTGC	-	Not submitted	-	-	-	57	2	62	236	Unselected
	China	Missense	604G>A	V202I	Benign	Not studied	Tolerated	Benign	-	1	-	-	-
	China	Missense	1888C>G	L630V	Benign	Not studied	Damaging	Probably damaging	-	1	-	-	-
	China	Splice site	1320+7 A>G	-	Not submitted	-	-	-	-	1	-	-	-
Richards [41]	Ireland	Splice site	49-2A>G	-	Pathogenic	-	-	-	?	1	?	8	HDGC
	Ireland	Non sense	59G>A	-	Pathogenic	-	-	-	-	1	-	-	-

Hansford [23]	Hispanic	Deletion	del 67328695-67328844	-	Not submitted	-	-	-	43	1	?	183	HDGC
Hansford [23]	Lithuania	Deletion	del 67324886-67330557	-	Not submitted	-	-	-	-	1	-	-	-
	Germany-Hung.	Deletion	del 67193822-67387415	-	Not submitted	-	-	-	-	1	-	-	-
	Eastern Europe	Deletion	1-?_163+?del	-	Not submitted	-	-	-	-	1	-	-	-
	Scotland-Irel.	Missense	3G>A	M1I	Likely pathogenic	Not studied	Damaging	Benign	-	1	-	-	-
	France-Canada	Splice site	48+1G>A	-	Pathogenic	-	-	-	-	1	-	-	-
	Italy	Missense	79C>T	P27S	VUS	Not studied	Tolerated	Benign	-	1	-	-	-
	Hispanic	Missense	286A>G	I96V	Likely benign	Not studied	Tolerated	Benign	-	1	-	-	-
	Lebanon	Deletion	382delC	-	Pathogenic	-	-	-	-	1	-	-	-
	UK	Deletion	447_453delCAGAAGA	-	Pathogenic	-	-	-	-	1	-	-	-
	Algeria	Splice site	687+1G>T	-	Not submitted	-	-	-	-	1	-	-	-
	Hispanic	Missense	715G>A	G239R	Likely pathogenic	Function loss	Damaging	Probably damaging	-	1	-	-	-
	Sweden-Germ.	Splice site	832+1G>T	-	Pathogenic	-	-	-	-	1	-	-	-
	Caucasian	Splice site	833-2A>G	-	Pathogenic	-	-	-	-	1	-	-	-
	Europe	Missense	892G>A	A298T	Benign	Function loss	Damaging	Probably damaging	-	1	-	-	-
	Mexico-Eur.	Non sense	940A>T	-	Pathogenic	-	-	-	-	1	-	-	-
	Caucasian	Splice site	1009-2A>G	-	Not submitted	-	-	-	-	1	-	-	-
	Germany	Splice site	1137G>A	-	Pathogenic	-	-	-	-	1	-	-	-
	America	Splice site	1137G>T	-	Likely pathogenic	-	-	-	-	1	-	-	-
	Unknown	Splice site	1137+1G>A	-	Likely pathogenic	-	-	-	-	1	-	-	-
	Caucasian	Non sense	1189A>T	-	VUS	-	-	-	-	1	-	-	-
	Japan	Deletion	1212delC	-	Pathogenic	-	-	-	-	1	-	-	-
	Sweden-UK	Splice site	1565+1G>A	-	Pathogenic	-	-	-	-	1	-	-	-
	Caucasian	Splice site	1565+1G>C	-	Pathogenic	-	-	-	-	1	-	-	-
	Arabia	Splice site	1565+1G>T	-	Pathogenic	-	-	-	-	1	-	-	-
	Unknown	Insertion	1565+2dupT	-	Pathogenic	-	-	-	-	1	-	-	-
	Caucasian	Missense	1679C>G	T560R	Pathogenic	Function loss	Damaging	Probably damaging	-	1	-	-	-
	Unknown	Non sense	1792C>T	-	Pathogenic	-	-	-	-	1	-	-	-
	Brasil	Deletion	2058_2059delTG	-	Not submitted	-	-	-	-	1	-	-	-
	UK-Scotland	Deletion	2100delT	-	Pathogenic	-	-	-	-	1	-	-	-
	Unknown	Non sense	2265T>A	-	Pathogenic	-	-	-	-	1	-	-	-
	Unknown	Deletion	2310delC	-	Pathogenic	-	-	-	-	1	-	-	-
Hansford [23]	Jewish	Missense	2413G>A	D805N	Benign	Function loss	Damaging	Probably damaging	-	1	-	-	-
	UK	Deletion	2430delT	-	Pathogenic	-	-	-	-	1	-	-	-
Shinmura [42]	Japan	Missense	185G>T	G62V	VUS	Function loss*	Damaging	Probably damaging	61	1	?	13	Unselected
Benusiglio [43]	France	Non sense	1147C>T	-	Pathogenic	-	-	-	40	1	?	162	Unselected
	France	Deletion	Del exon 11	-	Not submitted	-	-	-	-	1	-	-	-
	France	Splice site	832+1G>T	-	Pathogenic	-	-	-	-	2	-	-	-

	France	Deletion	2398delC	-	Pathogenic	-	-	-	-	1	-	-	-
	France	Insertion	1565+2dup	-	Pathogenic	-	-	-	-	1	-	-	-
	France	Splice site	1137G>A	-	Pathogenic	-	-	-	-	1	-	-	-
	France	Deletion	1470–1483del	-	Not submitted	-	-	-	-	2	-	-	-
	France	Splice site	1679C>G	-	Pathogenic	-	-	-	-	1	-	-	-
	France	Deletion	469delG	-	Not submitted	-	-	-	-	1	-	-	-
	France	Deletion	2386delC	-	Pathogenic	-	-	-	-	1	-	-	-
	France	Non sense	283C>T	-	Pathogenic	-	-	-	-	1	-	-	-
	France	Missense	2343A>T	E781D	VUS	Function loss	Tolerated	Benign	-	1	-	-	-
	France	Non sense	1595G>A	-	Pathogenic	-	-	-	-	1	-	-	-
Yamada [44]	Japan	Deletion	1212delC	-	Pathogenic	-	-	-	26	2	?	13	HDGC
	Japan	Deletion	164-?_387+?del	-	Not submitted	-	-	-	-	1	-	-	-
Brooks-Wilson [20]	Europe	Splice site	687+1G>A	-	Likely pathogenic	-	-	-	38	1	45	35	HDGC
	Caucasian	Insertion	1779dupC	-	Not submitted	-	-	-	-	1	-	-	-
	Unknown	Deletion	2310delC	-	Pathogenic	-	-	-	-	1	-	-	-
	Unknown	Missense	1225T>C	W409R	Not submitted	Function loss	Damaging	Probably damaging	-	1	-	-	-
	Caucasian	Deletion	1212delC	-	Pathogenic	-	-	-	-	1	-	-	-
	Italy	Deletion	382delC	-	Pathogenic	-	-	-	-	1	-	-	-
	Europe	Splice site	1711+5G>A	-	Pathogenic	-	-	-	-	1	-	-	-
	Turkey	Missense	2195G>A	R732Q	Likely pathogenic	Function loss	Damaging	Probably damaging	-	1	-	-	-
	USA	Deletion	2064_2065delTG	-	Not submitted	-	-	-	-	1	-	-	-
	Unknown	Deletion	1476_1477delAGdel	-	Not submitted	-	-	-	-	1	-	-	-
	Caucasian	Insertion	1064dupT	-	Not submitted	-	-	-	-	1	-	-	-
	Europe	Missense	892G>A	A298T	Benign	Function loss	Damaging	Probably damaging	-	1	-	-	-
	Caucasian	Deletion-insertion	1135_1137+5delinsTTAGA	-	Pathogenic	-	-	-	-	1	-	-	-
Suriano [45]	Portugal	Missense	1901C>T	A634V	Likely pathogenic	Function loss	Tolerated	Benign	41	1	<51	66	EOGC
	UK	Splice site	532-18 C>T	-	Benign/Likely benign	-	-	-	-	1	-	-	-
	Portugal	Splice site	532-18 C>T	-	Benign/Likely benign	-	-	-	-	1	-	-	-
Kim [46]	Korea	Missense	715G>A	G239R	Likely pathogenic	Function loss	Damaging	Probably damaging	38	1	<51	25	EOGC
	Korea	Non sense	1003C>T	-	Pathogenic	-	-	-	-	1	-	-	-
	Korea	Non sense	1003C>T	-	Pathogenic	-	-	-	-	1	-	-	-
Yoon [47]	Korea	Missense	731A>G	D244G	VUS	Not studied	Damaging	Probably damaging	44	1	41	5	HDGC
	Korea	Missense	1460T>C	V487G	Pathogenic	Not studied	Tolerated	Possibly damaging	-	1	-	-	-

Tsukanov [48]	Russia	Deletion	1005delA	-	Not submitted	-	-	-	?	1	?	9	Unselected
Kim [49]	Korea	Missense	1018A>G	T340A	Benign	Function loss	Damaging	Benign	30	6	?	131	Unselected
Zhang [50]	China	Missense	1018A>G	T340A	Benign	Function loss	Damaging	Benign	?	2	?	101	Unselected
Wang [51]	Japan	Missense	1243A>C	I415L	VUS	Unconclusive	Damaging	Benign	?	3	?	101	Unselected
Keller [52]	Germany	Deletion	372delC	-	Not submitted	-	-	-	41	1	?	35	HDGC
	Germany	Missense	2396C>G	P799R	VUS	Function loss	Damaging	Probably damaging	-	1	-	-	-
	Germany	Insertion	1619dupG	-	Pathogenic	-	-	-	-	1	-	-	-
Ascano [53]	USA	Missense	1849G>A	A617T	Benign	Retains function	Tolerated	Benign	64	1	?	40	Unselected
Yabuta [54]	Japan	Missense	2494G>A	V832M	Benign	Function loss	Damaging	Probably damaging	50	1	55	17	HDGC
El-Husny [55]	Brasil	Nonsense	1023T>G		Pathogenic	-	-	-	33	2	?	6	HDGC
Humar [56]	Caucasian	Deletion	53delC	-	Pathogenic	-	-	-	41	1	45	10	HDGC
	Arabia	Splice site	1565+1G>T	-	Pathogenic	-	-	-	-	1	-	-	-
	Maori	Non sense	1792C>T	-	Pathogenic	-	-	-	-	1	-	-	-
	Caucasian	Splice site	2295+5G>A	-	Not submitted	-	-	-	-	1	-	-	-
	Africa-Am.	Deletion	1710delT	-	Pathogenic	-	-	-	-	1	-	-	-
Roviello [57]	Italy	Missense	1118C>T	P373L	Likely benign	Function loss	Damaging	Probably damaging	79	1	65	14	HDGC

Table S2. *CDH1* germline mutations identified in Family Study group).

First Author	Country	Type	HGVS	Protein Change	ClinVar/LOVD Class.	In Vitro Data	SIFT	PolyPhen-2	Age Proband	CDH1m TOT	CDH1m KG	Mean Age CDH1mKG	CDH1m OtherK	Mean Age CDH1m OtherK	CDH1wt
Norero [58]	Chile	Non sense	1531C>T	-	Pathogenic	-	-	-	22	6	2	21	0	0	2
Katona [59]	USA	Splice site	1566-2A>G	-	Likely pathogenic	-	-	-	43	5	1	43	1	75 (PR)	0
Obermair [60]	Austria	Splice site	687+1G > A	-	Likely pathogenic	-	-	-	?	5	3	46	0	0	5
Pena-Couso [61]	Spain	Missense	1679C>G	T560R	Pathogenic	Function loss	Damaging	Probably damaging	?	8	3	36	0	0	1
	Spain	Missense	1679C>G	T560R	Pathogenic	Function loss	Damaging	Probably damaging	?	2	2	28	0	0	2
	Spain	Missense	1679C>G	T560R	Pathogenic	Function loss	Damaging	Probably damaging	33	8	2	41	0	0	4
Gullo [62]	Portugal	Missense	1901C>T	A634V	Likely pathogenic	Function loss	Tolerated	Benign	18	15	7	33	1	45 (LBR)	15
Caggiari [63]	Italy	Deletion	1612delG	-	Pathogenic	-	-	-	41	2	1	41	0	0	0
Betés [64]	Spain	Missense	977T>A	I326N	Not submitted	Function loss	Damaging	Probably damaging	44	3	3	48	0	0	0
Yelskaya [65]	USA	Splice site	1679C>G	-	Pathogenic	-	-	-	50	3	3	52	0	0	0
López [66]	Spain	Deletion	1220_1220delC	-	Not submitted	-	-	-	47	3	2	35	1	49 (CO)	3
Zhang [67]	Hispanic	Missense	48G>C	Q16H	Not submitted	Function loss	Damaging	Probably damaging	22	2	2	41	0	0	0
Sugimoto [68]	Japan	Deletion	1566-?_1711??del	-	Not submitted	-	-	-	41	2	1	41	0	0	2
Yamada [69]	Japan	Deletion	Large del exons 7–16	-	Not submitted	-	-	-	34	2	2	32	0	0	0
Bardram [70]	Denmark	Deletion	602_603delCT	-	Not submitted	-	-	-	32	4	1	32	0	0	2
	Denmark	Insertion	1565+3insTT	-	Pathogenic	-	-	-	44	12	5	53	1	27 (CA)	9
More [71]	Caucasian	Splice site	49-2A>C	-	Pathogenic	-	-	-	42	4	3	55	1	55 (CO)	1
	Caucasian	Missense	353C>G	T118R	VUS	Function loss	Tolerated	Benign	55	4	1	55	0	0	2
	Caucasian	Missense	715G>A	G239R	Likely pathogenic	Function loss	Damaging	Probably damaging	30	2	1	30	0	0	2
	Hispanic	Deletion	1107delC	-	Pathogenic	-	-	-	48	1	1	48	0	0	0
	Caucasian	Splice site	1137G>A	-	Pathogenic	-	-	-	29	4	2	29	1	36 (TO)	2
	Caucasian	Deletion	1391_1392delTC	-	Pathogenic	-	-	-	34	1	1	34	0	0	0
	Maori	Missense	1901C>T	A634V	Likely pathogenic	Function loss	Tolerated	Benign	35	1	1	35	0	0	0
	China	Non sense	2095C>T	-	Pathogenic	-	-	-	39	1	1	39	0	0	0
	Caucasian	Splice site	2440-6C>G	-	Benign/Likely benign	-	-	-	36	1	1	36	0	0	3
Kluijt [72]	Unknown	Deletion	55_74del20	-	Not submitted	-	-	-	27	1	1	27	0	0	0
	Unknown	Non sense	187C>T	-	Pathogenic	-	-	-	41	3	1	41	0	0	0
	Unknown	Non sense	489C>A	-	Pathogenic	-	-	-	?	3	2	49	1	44 (LBR)	0

	Hindustan	Deletion-ins	811_812delins12	-	Not submitted	-	-	-	55	4	2	55	0	0	1
	Unknown	Deletion-ins	1135_1137+5delins	-	Pathogenic	-	-	-	?	2	1	40	0	0	0
Kluijt [72]	Unknown	Deletion	1404delC	-	Not submitted	-	-	-	?	11	9	43	0	0	21
	Unknown	Deletion	1476_1477delAG	-	Pathogenic	-	-	-	42	2	1	42	0	0	0
	Unknown	Splice site-ins	1565+2dupT	-	Pathogenic	-	-	-	?	16	14	47	0	0	9
	Creole	Missense	1748T>G	L583R	Pathogenic	Function loss	Damaging	Probably damaging	40	2	2	29	0	0	0
	Turkey	Missense	2195G>A	R732Q	Likely pathogenic	Function loss	Damaging	Probably damaging	38	3/4	1/2	38	0	0	0
Guilford [73]	Europe	Insertion	1588insC	-	Pathogenic	-	-	-	40	3	3	50	0	0	0
	Europe	Non sense	70G>T	-	Pathogenic	-	-	-	?	6	3	43	1	39 (BR)	1
	Africa-Americ	Splice site	1137+1G>A	-	Likely pathogenic	-	-	-	25	3	3	25	0	0	0
	Europe	Non sense	586G>T	-	Pathogenic	-	-	-	31	1	1	31	0	0	0
	Maori	Non sense	190C>T	-	Pathogenic	-	-	-	22	1	1	22	0	0	0
	Unknown	Frameshift	1487del7	-	Pathogenic	-	-	-	31	1	1	31	0	0	0
Dussaulx-Garin [74]	France	Non sense	283C>T	-	Pathogenic	-	-	-	?	3	3	44	0	0	0
Keller [75]	Germany	Deletion	377delC	-	Pathogenic	-	-	-	38	2	2	48	1	49 (LBR)	1
Frebourg [76]	Caucasian	Splice site	531+2T>A	-	Pathogenic	-	-	-	23	4	3	32	1	23 (CA)	2
Norton [77]	USA	Non sense	1003C>T	-	Pathogenic	-	-	-	?	14	11	51	0	0	9
Rogers [78]	USA	Insertion	1565+2insT	-	Pathogenic	-	-	-	?	3	3	57	0	0	2
	USA	Deletion	2395delC	-	Not submitted	-	-	-	42	4	1	42	0	0	5
Guilford [79]	Maori	Splice site	1008G>T	-	Likely pathogenic	-	-	-	?	39	9	36	1	74 (CO)	0
	Maori	Non sense	2095C>T	-	Pathogenic	-	-	-	30	1	1	30	0	0	0
	Maori	Frameshift	2386dupC	-	Pathogenic	-	-	-	?	3	2	26	0	0	0
Oliveira [38]	Portugal	Deletion-ins	1135_1137+5delins	-	Pathogenic	-	-	-	52	1	1	53	0	0	1
	Portugal	Missense	1901C>T	A634V	Likely pathogenic	Function loss	Tolerated	Benign	23	1	1	23	0	0	0
Mayrbaeurl [80]	Austria	Deletion-ins	1304_1305insA	-	Pathogenic	-	-	-	49	10	7	Unknown	0	0	1
Jiang [81]	China	Non sense	1507C>T	-	Pathogenic	-	-	-	48	1	1	48	0	0	4
Rodriguez-Sanjuan [82]	Spain	Deletion	1610delC	-	Pathogenic	-	-	-	58	9	3	60	0	0	0
Shah [83]	USA	Non sense	1792C>T	-	Pathogenic	-	-	-	25	2	2	38	0	0	3
Van Domselaar [84]	Spain	Non sense	1913G>A	-	Pathogenic	-	-	-	28	1	1	28	0	0	0
Ghaffari [85]	Iran	Non sense	2275G>T	-	Pathogenic	-	-	-	37	2	0	37	0	0	2
	Europe	Non sense	187C>T	-	Pathogenic	-	-	-	?	5	2	Unknown	0	0	2

	Europe	Insertion	1711+1dupG	-	Pathogenic	-	-	-	?	3	3	51	0	0	3
	Europe	Non sense	1792C>T	-	Pathogenic	-	-	-	?	2	2	25	0	0	0
Kaurah [19]	Unknown	Non sense	283C>T	-	Pathogenic	-	-	-	?	1	1	Unknown	0	0	0
	Filipine	Missense	715G>A	G239R	Likely pathogenic	Function loss	Damaging	Probably damaging	?	1	1	Unknown	0	0	0
	Unknown	Splice site	1137G>A	-	Pathogenic	-	-	-	?	1	1	-	0	0	0
	Italy	Splice site	1137G>A	-	Pathogenic	-	-	-	?	2	2	-	0	0	0
	Sweden	Splice site	1137G>A	-	Pathogenic	-	-	-	?	1	1	-	0	0	0
	UK	Deletion	1397-1398delTC	-	Pathogenic	-	-	-	?	1	1	-	0	0	0
	Ireland	Insertion	1682dupA	-	Pathogenic	-	-	-	?	1	1	-	0	0	0
	UK	Missense	1901C>T	A634V	Likely pathogenic	Function loss	Tolerated	Benign	?	2	2	-	0	0	0
	Portugal	Missense	1901C>T	A634V	Likely pathogenic	Function loss	Tolerated	Benign	?	1	1	-	0	0	0
	Portugal	Missense	1901C>T	A634V	Likely pathogenic	Function loss	Tolerated	Benign	?	1	1	-	0	0	0
	Spanish	Non sense	1913G>A	-	Pathogenic	-	-	-	?	1	1	-	0	0	0
	Germany	Deletion	2064-2065delTG	-	Pathogenic	-	-	-	?	2	2	-	0	0	0
	UK-Scotland	Deletion	2064-2065delTG	-	Pathogenic	-	-	-	?	1	1	-	0	0	0
	Unknown	Splice site	2164+5G>A	-	Not submitted	-	-	-	?	1	1	-	0	0	0
	Unknown	Missense	2195G>A	R732Q	VUS	Function loss	Damaging	Probably damaging	?	1	1	-	0	0	0
	UK	Missense	2195G>A	R732Q	VUS	Function loss	Damaging	Probably damaging	?	3	3	-	0	0	0
	Colombia	Missense	2245C>T	R749W	VUS	Function loss	Damaging	Probably damaging	?	1	1	-	0	0	0
	UK	Missense	2343A>T	E781D	VUS	Function loss	Tolerated	Benign	?	1	1	-	0	0	0
	France	Deletion	2398delC	-	Pathogenic	-	-	-	?	?	2	?	?	?	?
	UK-Ireland	Deletion	2398delC	-	Pathogenic	-	-	-	39	?	1	39	?	?	?
	UK-Ireland	Deletion	2398delC	-	Pathogenic	-	-	-	34	?	2	?	?	?	?
	UK-Ireland	Deletion	2398delC	-	Pathogenic	-	-	-	53	?	4	?	?	?	?
Oliveira [86]	Pakistan	Splice site	832G>A	-	Pathogenic	-	-	-	?	4	3	37	1	LBR (Unknown)	0
	Europe	Missense	1018A>G	T340A	Benign	Function loss	Damaging	Benign	47	1	1	47	0	0	0
	Europe	Insertion	1472dupA	-	Pathogenic	-	-	-	30	1	1	30	0	0	0
	Europe	Insertion	44dupT	-	Pathogenic	-	-	-	59	4	1	30	3	CO(70,59) LBR (70)	0
Caron [87]	France	Deletion	2399delC	-	Pathogenic	-	-	-	45	7	6	31	0	0	8
Black [88]	Hawaii	Non sense	trp20stop	-	Not submitted	-	-	-	22	1	1	22	0	0	0
Oliveira [89]	Europe	Deletion	1135_1137+5delins	-	Pathogenic	-	-	-	27	4	4	35	0	0	2
Lynch [90]	USA	Non sense	1003C>T	-	Pathogenic	-	-	-	58	12	11	59	0	0	8
-	USA	Non sense	70G>T	-	Pathogenic	-	-	-	51	12	5	41	3	BR (57,46,39)	11

Lynch [90]	USA	Missense	2195G>A	R732Q	Likely pathogenic	Function loss	Damaging	Probably damaging	58	8	3	46	0	0	4
-	USA	Non sense	1792C>T	-	Pathogenic	-	-	-	42	6	1	61	1	BR (58)	3

"?" means "Unknown".

Table S3. *CDH1* germline mutations identified in Unknown Study group (*Unpublished data).

First Author	Country	Type	HGVS	Protein Change	ClinVar/LOVD Classification	In Vitro Data	SIFT	PolyPhen-2
Guilford* [79]	Unknown	Missense	2T>C	M1T	Not submitted	Not studied	Damaging	Possibly damaging
	Unknown	Insertion	46_48insTGC	-	Not submitted	-	-	-
	Unknown	Non sense	1023T>G	-	Pathogenic	-	-	-
	Unknown	Insertion	753insG	-	Not submitted	-	-	-
	Unknown	Non sense	2287G>T	-	Pathogenic	-	-	-
Caldas-Hansford* [23]	Unknown	Non sense	67C>T	-	Not submitted	-	-	-
	Europe	Missense	641T>C	L214P	Pathogenic	Function loss	Damaging	Probably damaging
	Caucasian	Splice site	1009-2delAG	-	Not submitted	-	-	-
	Arabia	Splice site	1137G>A	-	Pathogenic	-	-	-
	UK	Insertion	1466dupC	-	Pathogenic	-	-	-
	Unknown	Deletion	1733delC	-	Not submitted	-	-	-
	UK-Scotland	Deletion	2100delT	-	Pathogenic	-	-	-
	Unknown	Missense	2248G>A	D750N	VUS	Unconclusive	Damaging	Probably damaging
	UK	Deletion	2400delG	-	Not submitted	-	-	-
Simoes-Correia [91]	Portugal	Missense	641T>C	L214P	Pathogenic	Function loss	Damaging	Probably damaging
	Portugal	Missense	554A>T	E185V	VUS	Retains function	Tolerated	Benign
	Portugal	Missense	695C>G	S232C	VUS	Retains function	Damaging	Probably damaging
Charlton [92]	USA	Non sense	2287G>T	-	Pathogenic/Likely pathogenic	-	-	-
van der Post [21]	Unknown	Deletion	971del	-	Not submitted	-	-	-
	Unknown	Splice site	2439+5_2439+8 delGTAA	-	Not submitted	-	-	-

Copyright: © 2021 by the author. Licensee MDPI, Basel, Switzerland. This article is an open access article distributed under the terms and conditions of the Creative Commons Attribution (CC BY) license (http://creativecommons.org/licenses/by/4.0/).
